# Supplementary material for: Causal interplay between lactose intolerance and gut microbiota: a combined bidirectional Mendelian randomization and in vivo validation study
Source: Front Nutr. 2026 Jun 1;13:1803337. doi: 10.3389/fnut.2026.1803337 (PMC13265576; doi:10.3389/fnut.2026.1803337)
Supplement: Supplementary file 1 [file Data_Sheet_1.zip › supplementary materials/Forward/leave one out cross validation/ebi-a-GCST90027611.finngen_R12_E4_LACTONAS.pdf]

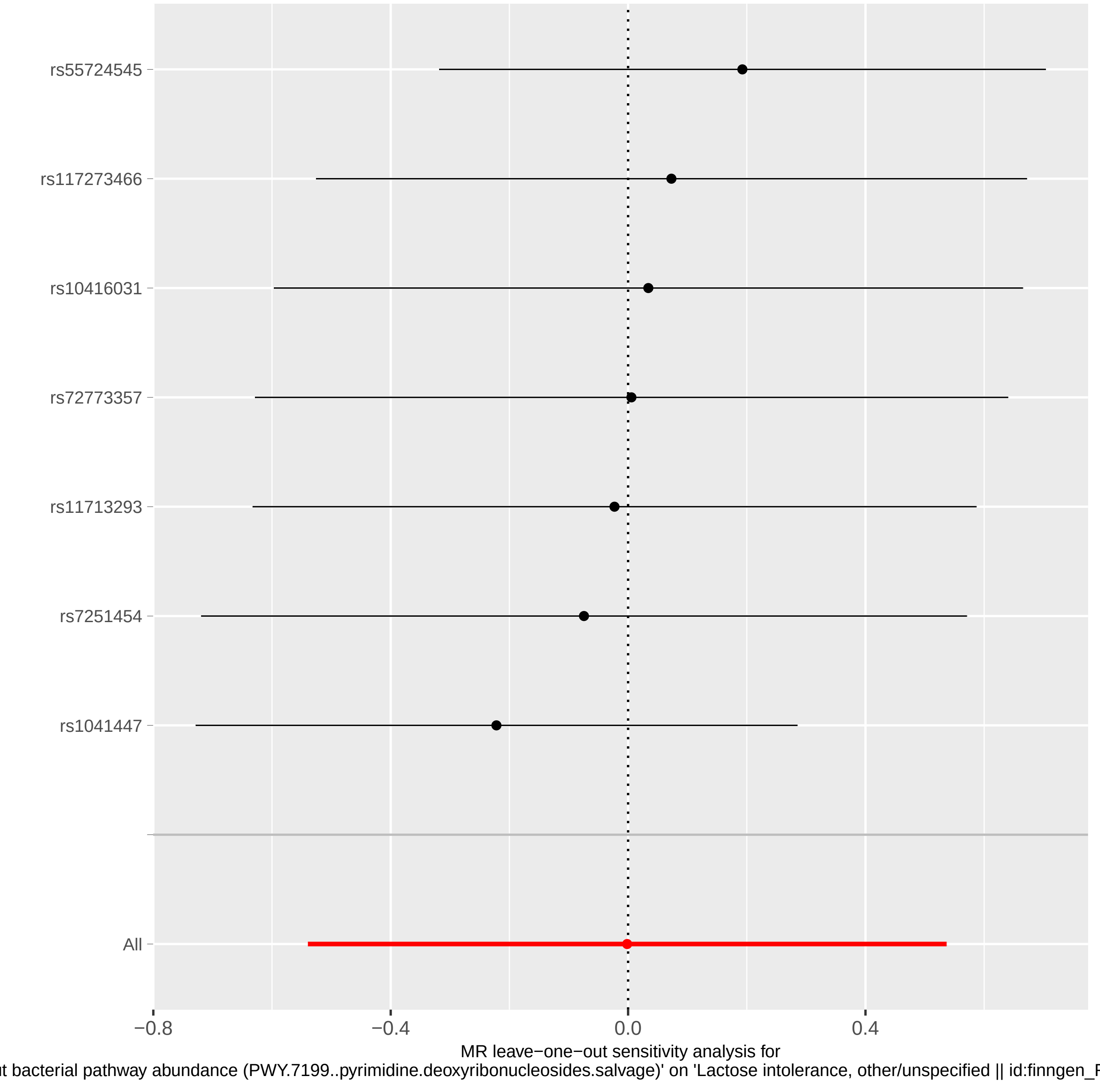

rs55724545
rs117273466
rs10416031
rs72773357
rs11713293
rs7251454
rs1041447
All
−0.8
−0.4
0.0
0.4
MR leave−one−out sensitivity analysis for
t bacterial pathway abundance (PWY.7199..pyrimidine.deoxyribonucleosides.salvage)' on 'Lactose intolerance, other/unspecified || id:finngen_F
